# Predictors of response to pegylated interferon in chronic hepatitis B: a real-world hospital-based analysis

**DOI:** 10.1038/srep29605

**Published:** 2016-07-12

**Authors:** Yin-Chen Wang, Sien-Sing Yang, Chien-Wei Su, Yuan-Jen Wang, Kuei-Chuan Lee, Teh-Ia Huo, Han-Chieh Lin, Yi-Hsiang Huang

**Affiliations:** 1 Division of Gastroenterology and Hepatology, Department of Medicine, Taipei Veterans General Hospital, Taipei, Taiwan; 2 Liver Center, Cathay General Hospital Medical Center, Taipei, Taiwan; 3 Health Care Center, Taipei Veterans General Hospital, Taipei, Taiwan; 4 Institute of Pharmacology, National Yang-Ming University School of Medicine, Taipei, Taiwan; 5 Institute of Clinical Medicine, National Yang-Ming University School of Medicine, Taipei, Taiwan

## Abstract

Information on the efficacy of pegylated interferon (PEG-IFN) treatment of chronic hepatitis B (CHB) patients and predictors of the response based on real-world data is limited. Consecutive 201 patients who underwent PEG-IFN treatment for CHB were reviewed. A virological response (VR) was defined as a serum HBV DNA of <2000 IU/mL, and a combined response (CR) was defined a VR accompanied by serological response for hepatitis B e antigen (HBeAg)-positive CHB. For HBeAg-positive CHB patients, the HBeAg seroconversion rate and CR rate were 30.5% and 21.2% at 48 weeks after end of treatment (EOT), respectively. Baseline alanine aminotransferase (ALT) level was associated with HBeAg seroconversion, while baseline hepatitis B s antigen (HBsAg) levels of <250 IU/mL and HBV DNA <2.5 × 10^7^ IU/mL were strongly associated with sustained off-treatment CR. For HBeAg-negative CHB, the VR rates were 85.5%, and 27.7% at EOT, and 48 weeks after EOT, respectively; a baseline HBsAg <1,250 IU/mL was associated with sustained off-treatment VR. PEG-IFN treatment has durable HBeAg seroconversion in HBeAg-positive CHB, but results in a high risk of relapse among HBeAg-negative CHB patients. Pre-treatment HBsAg level is an important predictor of VR in CHB patients undergoing PEG-IFN treatment.

Hepatitis B virus (HBV) infection is a global health issue. Approximately, 248 million people are chronic carriers of the HBV across the world^1^. Chronic HBV infection may result in the development of serious complications, such as liver cirrhosis and hepatocellular carcinoma (HCC). Although a hepatitis B vaccine has been available for more than two decades, there is still between 5% and 10% of the adult population chronically infected with HBV in Asia^2^,^3^.

The treatment options for chronic hepatitis B include nucleoside or nucleotide analogues (NUCs), and interferon (IFN) or pegylated interferon (PEG-IFN) based on current treatment guidelines^4^,^5^,^6^. NUCs are able to effectively suppress the HBV viral loads and reduce liver-related morbidity and mortality^7^,^8^,^9^. However, the virological and clinical relapse rates are high after cessation of the treatment; therefore long-term treatment is usually required^10^. In contrast, PEG-IFN has the advantage of a finite course treatment duration without the possibility of drug resistance. Nevertheless, the response rate of PEG-IFN is not satisfactory based on previous clinical trials^11^,^12^,^13^. Due to the more frequent adverse effects and the route of administration, only limited numbers of CHB patients have received PEG-IFN treatment in clinical practice, consequently, real-world data on the response to PEG-IFN among CHB patients has rarely been reported.

Several prediction factors are known to be associated with treatment response among PEG-IFN treated patients based on previous clinical trials. In HBeAg-positive CHB patients, a high alanine aminotransferase (ALT) level, a low HBV viral load at baseline, genotype A HBV, and a decline in serum HBsAg level during the treatment have been associated with a clinical response in previous studies^14^,^15^,^16^,^17^. Among HBeAg-negative CHB patients, very few baseline predictors have been proposed and a response-guided treatment according to serial HBsAg decline is recommended^18^,^19^,^20^,^21^,^22^,^23^. Nevertheless, predictors of PEG-IFN treatment among CHB patients based on real-world data remains in an unmet need.

The aim of this study was to analyze the factors associated with a sustained off-treatment response to PEG-IFN α2a based treatment using a real-world cohort of CHB patients from two medical centers in Taiwan, and to develop a model for identifying patients who have the best chance of responding to PEG-IFN treatment in clinical practice.

## Patients and Methods

### Patients

Consecutive CHB patients who underwent and completed PEG-IFN α2a treatment from 2005 to 2013 were retrospectively reviewed from Taipei Veterans General Hospital and Cathay General Hospital. The inclusion criteria were age ≥20 years old, positive for hepatitis B surface antigen (HBsAg) for more than 6 months before the treatment with either a positive or negative status for HBeAg, a pretreatment serum alanine aminotransferase (ALT) equal to or more than 2 times the upper limit of normal (ULN), a baseline HBV viral load of ≥20,000 IU/mL for HBeAg-positive cases and of ≥2,000 IU/mL for HBeAg-negative cases. The exclusion criteria included hepatitis C or hepatitis D virus co-infection, associated hepatitis related to autoimmune disease or alcohol, the patient having cirrhosis or HCC at baseline, incomplete medical records regarding the treatment response at EOT and 48 weeks after EOT, and a treatment course of less than 3 months due to intolerance or adverse events. This study was approved by Institutional Review Board Taipei Veterans General Hospital, and Institutional Review Board of the Cathay General Hospital. As a retrospective cohort data, patient informed consent was waived by both Institutional Review Boards.

### PEG-IFN treatment duration and definition of responses to therapy

Based on the regulations of Taiwan National Health Insurance (NHI), adult CHB patients were eligible for PEG-IFN treatment when fulfilling the following criteria: (1) positive for HBsAg for at least 6 months (2) for HBeAg-positive patients, elevation of serum ALT levels to at least two times that of the ULN, combined with a HBV DNA level of ≥20,000 IU/mL, and no liver decompensation; these cases are able to receive 24 weeks PEG-IFN treatment; (3) for HBeAg-negative patients, elevation of serum ALT levels to two times that of the ULN at least twice and at least 3 months interval apart, combined with a HBV viral load of ≥2,000 IU/mL; these cases are able to receive 48 weeks of Peg-IFN treatment.

Patients received PEG-IFN α2a 180 μg once weekly for 24 or 48 weeks for HBeAg-positive and HBeAg-negative CHB, respectively, and the dosage was adjusted according to any adverse effect identified by their physician. The primary outcome was a sustained off-treatment virological response (VR) as defined by the EASL treatment guideline (serum HBV DNA of <2000 IU/mL at 48 weeks post-treatment) for all CHB patients; and a serological response (HBeAg seroconversion) for HBeAg-positive CHB patients^24^. A combined response (CR) was defined as both a serological response and a VR for HBeAg-positive patients. Responses after EOT were defined as delayed response. The secondary outcome was the relapse rate. Relapse was defined as a HBV viral rebound to higher than 2000 IU/mL among the virological responders or e seroreversion among the serological responders who were HBeAg-positive CHB patients.

### Serological and biochemical assays

Routine complete blood counts and biochemical tests were performed using a systemic multi-autoanalyzer (Technicon SMAC, Technicon Instruments Corp., Tarrytown, NY) at baseline and monthly during therapy. The serum HBeAg and anti-HBe antibody levels were measured by radio-immunoassay (Abott Laboratories, North Chicago, IL). HBsAg was quantified by Elecsys HBsAg II assay (Roche Diagnostics GmbH, Mannheim, Germany). HBV viral load was determined by a PCR-based method, namely the Roche Cobas Taqman HBV DNA assay (detection limit of 12 IU/mL, Roche Diagnostics, Switzerland).

### Statistical analysis

Patients were categorized via a number of different approaches. Pearson chi-square analysis or Fisher exact test was used to compare categorical variables, while the Student t test or Mann-Whitney U test was used to compare continuous variables. The associations between parameters at baseline, on-treatment, and post-treatment with response were examined by logistic regression analyses. Variables in each period with *p* < 0.1 were further analyzed using a multivariate logistic regression model to identify independent variables that predicted a response. The models were developed to predict sustained off treatment responses based on the univariate and multivariate analyses. Odds ratios (OR) were convert to integer scores and the score might be adjusted according to the p value. All statistical analyses were performed using the Statistical Package for Social Sciences (SPSS 17.0 for Windows, SPSS Inc, Chicago, IL). A 2-tailed *p* value <0.05 was considered significant.

## Results

### Characteristics of the patients

A total of 222 CHB patients (126 HBeAg-positive, 96 HBeAg-negative) were reviewed from 2005 to 2013. By exclusion criteria, 201 CHB patients (118 HBeAg-positive, 83 HBeAg-negative) were enrolled (Table 1). HBeAg-positive CHB patients were significantly younger, and had higher HBV DNA levels and a higher proportion with high HBV viral loads (≥2.5 × 10^7^ IU/mL). There were no differences in gender, baseline ALT level, and HBsAg level between the two groups.

### Serological, virological and combined responses

**Figure 1:**
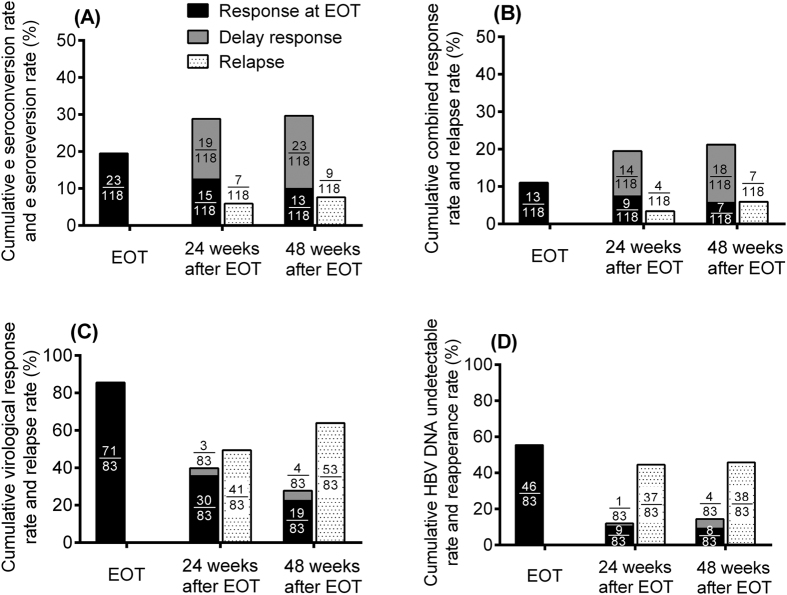
Off-treatment responses among HBeAg-positive and HBeAg-negative chronic hepatitis B patients. (**A**) Cumulative HBe seroconversion rate and HBe seroreversion rate among HBeAg-positive CHB patients; (**B**) Cumulative combined response rate and relapse rate among HBeAg-positive CHB patients; (**C**) Cumulative virological response rate and relapse rate among HBeAg-negative CHB patients; (**D**) Cumulative HBV DNA undetectable rate and relapse rate among HBeAg-negative CHB patients (CHB, chronic hepatitis B; HBV, hepatitis B virus; EOT, End of treatment). *The black bar means patients who had already achieved response [either HBeAg seroconversion (Fig. 1A), CR (Fig. 1B), VR (Fig. 1C) or HBV viral load undetectable (Fig. 1D)] at EOT. The “delay response” (grey bar) indicated patients achieving response [either HBeAg seroconversion (Fig. 1A), CR (Fig. 1B), VR (Fig. 1C) or HBV viral load undetectable (Fig. 1D)] after EOT.

Among the 118 HBeAg-positive CHB patients, HBeAg seroconversion rates were 19.5% (n = 23) and 30.5% (n = 36) at EOT and 48 weeks after EOT (Fig. 1A). The ALT normalization rates were 38% (n = 45) and 45% (n = 53); and the combined response rates were 11% (n = 13) and 21.2% (n = 25) at EOT and 48 weeks after EOT, respectively (Fig. 1B). The median HBV DNA reduction at EOT and 48 weeks post EOT were both -2.6 log IU/mL as compared with the baseline levels.

Among the 83 HBeAg-negative patients, the virological response rates were 85.5% (n = 71) and 27.7% (n = 23) (Fig. 1C) and the ALT normalization rate were 45% (n = 37) and 49% (n = 41) at EOT and 48 weeks after EOT, respectively. The HBV DNA undetectable rates (HBV DNA <12 IU/mL) were 55.4% (n = 46), and 13.3% (n = 11) at EOT, and 48 weeks after EOT, respectively (Fig. 1D). The median reductions in HBV DNA were −4.3 log IU/mL at EOT and −1.1 log IU/mL at 48 weeks after EOT as compared with the baseline levels.

### HBsAg loss

Within 48 weeks after EOT, only one case among the HBeAg-positive patients and two cases among the HBeAg-negative patients developed HBsAg loss and none had anti-HBs seroconversion.

### Factors associated with responses in HBeAg-positive CHB patients

Baseline factors associated with the e seroconversion and the sustained off-treatment combined response among HBeAg-positive CHB patients were analyzed (Table 2). By univariate analysis, the baseline ALT ≥200 IU/L and HBsAg <25,000 IU/mL were associated with e seroconversion among HBeAg-positive patients. After multivariate analysis, baseline ALT ≥200 IU/L remained associated with e seroconversion. By univariate analysis, baseline HBV DNA <2.5 × 10^7^ IU/mL, ALT ≥200 U/L, and HBsAg <250 IU/mL were associated with the combined response among HBeAg-positive patients. After multivariate analysis, only baseline HBV DNA of <2.5 × 10^7^ IU/mL and HBsAg level of <250 IU/mL remained independently associated with the combined response among HBeAg-positive patients.

On-treatment HBsAg level on weeks 12 was available in 34 HBeAg-positive cases. Of them, 22 patients had at least 10% of decline compare to their pretreatment level. Genotype was available in 39 cases. Of them, 22 were genotype B, 17 were genotype C. Nether on-treatment HBsAg decline nor genotype of HBV was associated with response in the subgroup patients (Supplementary Table S1).

### Models for predicting PEG-IFN treatment response in HBeAg-positive CHB patients

**Figure 2:**
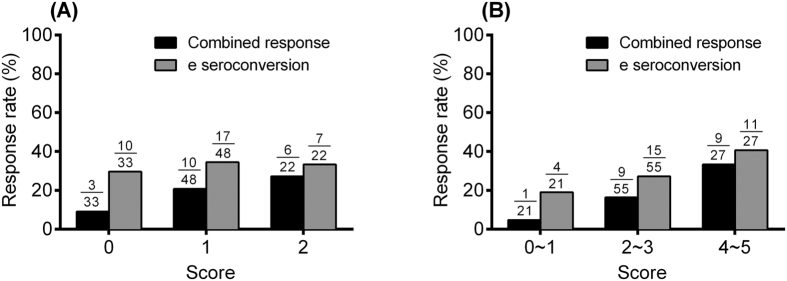
Response rates of models for a sustained off-treatment response at 48 weeks after end of treatment among HBeAg-positive chronic hepatitis B patients. The HBe seroconversion rate and combined response rate in HBeAg-positive chronic hepatitis B patients with different scores according to (**A**) model A, and (**B**) model B.

Based on the odds ratios and p value obtained from the multivariate analysis of responses among HBeAg-positive CHB patients, individual scores were assigned to each factor as listed in Table 3. Model A included in HBsAg <250 IU/mL and HBV DNA <2.5 × 107 (IU/mL). In the model B, we further enrolled baseline ALT ≥200 U/L and adjusted the weighted point; because the baseline ALT ≥200 U/L was significant in multivariate analysis of the e seroconversion and univariate analysis of the combined response for HBeAg-positive CHB patients. According to the scores, the response rates were categorized into three groups: a low, median, and high probability of response for HBeAg-positive patients (Fig. 2). Among HBeAg-positive patients, the sustained off-treatment combined response rate/and HBe seroconversion rates were 9% (3/33)/ 30% (10/33), 21% (10/48)/ 35% (17/48), and 27% (6/22)/ 32% (7/22) for the low probability group (score 0), median probability group (score 1), and high probability group (score 2) in model A. The sustained off-treatment combined response rate/and HBe seroconversion rates were 5% (1/21)/ 19% (4/21), 16% (9/55)/ 27% (15/55), and 33% (9/27)/ 41% (11/27) for the low probability group (score 0,1), median probability group (score 2,3), and high probability group (score 4,5) in model B.

### Factors associated with responses in HBeAg-negative CHB patients

**Figure 3:**
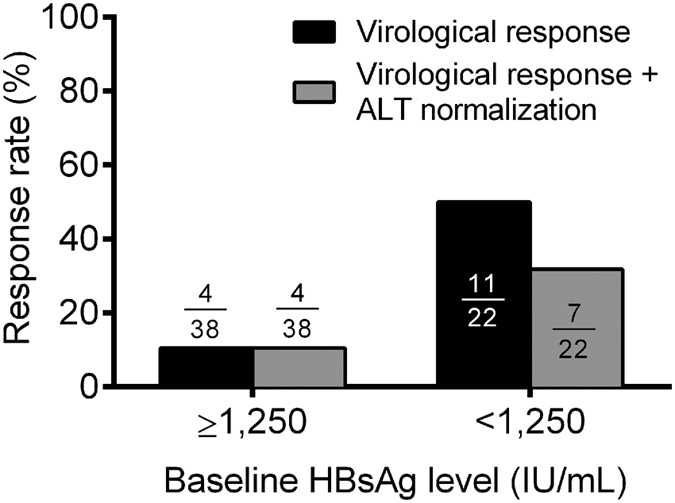
Response rates at 48 weeks after EOT among HBeAg-negative chronic hepatitis B patients. The response rates among HBeAg-negative chronic hepatitis B patients according to baseline HBsAg levels.

Factors associated with a sustained off-treatment VR among HBeAg-negative CHB patients were listed in Table 4. Among the HBeAg-negative CHB patients, the baseline HBsAg of <1,250 IU/mL, and the treatment-experienced were associated with sustained off-treatment response in univariate analysis. After multi-variate analysis, only a baseline HBsAg of <1,250 IU/mL was the significant one. The virological response rates at 48 weeks off-treatment were 50% (11/22) and 10.5% (4/38) in HBeAg-negative CHB patients with baseline HBsAg of <1,250 IU/mL and ≥1,250 IU/mL. (Fig. 3)

On-treatment HBsAg on week 12 were available in 35 HBeAg-negative CHB patients. On-treatment HBsAg decline ≥10% (OR: 8.75, p = 0.056) or ≥1 log (OR: 25.83, p = 0.004) were associated with sustained off-treatment response. Thirty-five patients had available HBV genotype data (23 genotype B, and 12 genotype C). There were no differences in response between genotype B and C patients by logistic regression analysis (Supplementary Table S2).

### Relapse

Among all HBeAg-positive patients, only 9 (7.6%) and 7 (5.93%) cases developed relapse after achieving serological or combined response at EOT (Fig. 1A,B). However, 53 (63.9%) HBeAg-negative CHB patients developed relapse at 48 weeks after EOT (Fig. 1C), and 38 cases (45.8%) became HBV DNA detectable at 48 weeks after EOT (Fig. 1D).

Among HBeAg-negative CHB patients, baseline HBsAg ≥1,250 IU/mL and detectable HBV viral load at EOT were significantly associated with relapse among responders at EOT by logistic regression analysis (Supplementary Table S3). Individual scores were assigned to each factor as listed in Supplementary Table S4. The model A included the baseline HBsAg ≥1,250 IU/mL and detectable HBV viral load at EOT. In subgroup of cases, on-treatment HBsAg decline <1 log and HBsAg ≥200 IU/mL at EOT were included into model B. According to the scores, the risk of relapse can be stratified into three groups (Supplementary Figure S1). Patients who fulfilled all factors in each model had the highest risk of relapse.

## Discussions

PEG-IFN is one of the main standard treatments for CHB, however, the efficacy of PEG-IFN, based on real-world data, has rarely been reported. PEG-IFN showed durable e seroconversion in HBeAg-positive CHB in our real-world data. Only few patients experienced HBeAg seroreversion, and an additional 20% of the patients would achieve delayed e seroconversion after completing the treatment. In the phase II and III studies of PEG-IFN, the HBeAg seroconversion rate was equivalent by 24 or 48 weeks of the treatment^13^,^25^. Therefore, 24 weeks of PEG-IFN remains the standard of care for HBeAg-positive CHB in Taiwan; even though 48 weeks of PEG-IFN treatment has been shown to provide a better e seroconversion rate than 24 weeks of treatment (36.2% *vs*. 22.9% at 6 months after EOT)^11^. The HBeAg seroconversion rate of 31.4% in our HBeAg-positive patients was similar to the report of the phase III study. The long-term effect of PEG-IFN is durable for HBeAg-positive CHB in our finding which complies with previous findings^11^,^13^,^26^.

A low HBV viral load and a high ALT level at baseline were associated with serological response of IFN treatment.^14^,^27^,^28^,^29^ Recently, on-treatment HBsAg decline is considered to be an important factor associated with the response to PEG-IFN in both HBeAg-positive and HBeAg-negative CHB^18^,^19^,^21^,^30^. An on-treatment HBsAg level of <1,500 IU/mL at weeks 12 or 24 was strongly associated with immune control in HBeAg-positive CHB^17^,^31^. HBsAg production is associated with HBV replication and the level of intrahepatic cccDNA^32^, but only few studies proposed the importance of pretreatment HBsAg level to predict PEG-IFN response^20^,^33^. HBsAg level is also a predictor when switching to PEG-IFN in HBeAg-positive CHB patients under long-term NUCs treatment^34^. The best cut-off for the baseline HBsAg level when predicting the combined response in HBeAg-positive CHB has not been well-established in the past. In our study, a baseline HBsAg level of <250 IU/mL had the highest sustained off-treatment CR rate of 30.3%. The limitation was that only 32% (33/103) of HBeAg-positive patients met this criterion. Among patients whose baseline HBsAg levels were between 250 IU/mL to 1,250 IU/mL, the sustained CR rate was 25%; and among patients with a high baseline HBsAg (≥1,250 IU/mL), the sustained CR rate was only 14.3%. Among the patients with available on-treatment HBsAg data in HBeAg-positive CHB patients, only few cases had achieved HBsAg decline and there was no effect on the response.

Previous studies have produced several models for predicting the response to PEG-IFN among HBeAg-positive CHB patients. Buster *et al.*^14^ suggests genotypes B and C patients who have high ALT levels and low HBV viral loads at baseline are likely to have the highest sustained response to PEG-IFN among HBeAg-positive CHB. A scoring model based on a HBV viral load of <2.5 × 10^7^ IU/mL, a HBsAg level of <250 IU/mL, with or without an ALT ≥200 U/L, was established to predict the response to PEG-IFN among HBeAg-positive CHB. This model has a number of advantages. Firstly, all of the parameters are baseline factors and thus can be applied before treatment begins. Secondly, these parameters are easily accessible in a real-world clinical situation without additional laboratory tests, except that the HBsAg quantification is not widely available in all countries.

Among HBeAg-negative patients, an extremely high VR at EOT was observed in our real-world data, but the relapse rate was also high. Marcellin *et al.*^35^ also reported that the virological response with a HBV viral load of <20,000 copies or <400 copies were 43%, and 19%, respectively at 6 months after 48 weeks of PEG-IFN treatment. These findings indicate that PEG-IFN is able to temporarily suppress HBV but the durability of the treatment is poor in HBeAg-negative CHB^22^,^36^. In our study, only a baseline HBsAg <1,250 IU/mL was able to predict a sustained off-treatment VR rate of 50% among HBeAg-negative CHB patients. The level of HBsAg may reflect the degree of host immune control in response to HBV, and a lower HBsAg might have a better outcome in the natural course of CHB^37^. Previous studies were not able to identify definite pretreatment factors that can predict the treatment response to PEG-IFN in HBeAg-negative cases. Our previous study suggested that a baseline CXCL9 level is associated with the response to PEG-IFN^16^. A further study to combine baseline HBsAg of <1250 IU/mL and a high CXCL9 level might improve the prediction rate for HBeAg-negative CHB in the future. On-treatment more than 10% of HBsAg decline is a sign of HBsAg loss in HBeAg-negative CHB under PFG-IFN treatment^30^. Our data also showed the same finding in the subgroup of HBeAg-negative patients with available on-treatment HBsAg data.

The poor durability of PEG-IFN treatment when treating HBeAg-negative CHB patients is still challenging. Patients who were treatment-experienced (17% *vs*. 48%, *p* = 0.007) had significant lower risk of relapse in univariate analysis only. Compared to the treatment-naive group, the treatment-experienced group had higher proportion with a HBsAg level <1250 IU/mL (33% *vs*. 65%, *p* = 0.01). Baseline HBsAg level, degree of on-treatment HBsAg decline, and detectable of HBV DNA at EOT are associated with the durability of the virological response to PEG-IFN among HBeAg-negative CHB patients.

There are several limitations in this study. Firstly, as a real-world retrospective study, the HBV genotype was not available in all patients, but genotype B and C seemed not associated with VR in subgroups of patients with available data. Secondly, on-treatment HBsAg levels were not available in some patients. However, on-treatment HBsAg is not an ideal predictor to select patients who are suitable for PEG-IFN before the treatment. Thirdly, our study was based on the standard of care in Taiwan with 24 weeks of PEG-IFN for HBeAg-positive CHB. Whether the model could be applied for HBeAg-positive cases with 48 weeks of PEG-IFN treatment needs further validation. Finally, further external validation is required for our prediction model.

In conclusion, PEG-IFN treatment should be carefully applied to selected CHB patients who have the best chance of a sustained off-treatment response. Pre-treatment HBsAg level is an important predictor for both HBeAg-positive and HBeAg-negative CHB for PEG-IFN treatment.

## Additional Information

**How to cite this article**: Wang, Y.-C. *et al.* Predictors of response to pegylated interferon in chronic hepatitis B: a real-world hospital-based analysis. *Sci. Rep.* **6**, 29605; doi: 10.1038/srep29605 (2016).

## Supplementary Material

Supplementary Information

## Figures and Tables

**Table 1:** Baseline characteristics of the 201 chronic hepatitis B patients undergoing PEG-IFN treatment.

	HBeAg-positive	HBeAg-negative	*p*
	N = 118	N = 83	
Age (y.)	37.9 ± 9.2	46.4 ± 9.7	<0.001
Male (%)	84 (71%)	67 (89%)	0.124
ALT (U/L)	222.4 ± 212.8	188.6 ± 177.5	0.237
AST (U/L)	110.6 ± 108.6	98.7 ± 91.8	0.334
Platelets (x10^3^/mm^3^)	196.4 ± 46.8	180.8 ± 65	0.069
HBsAg (IU/mL)*	1702 (46 ~ 291588)	1991 (34 ~ 50762)	0.113
HBV DNA (log IU/mL)	7 ± 1.4	5.6 ± 1.5	<0.001
HBV DNA ≥2.5 × 10^7^ IU/mL (%)	59 (50%)	11 (13%)	<0.001
Treatment duration (weeks)	26.7 ± 9	48.1 ± 1.8	<0.001
Treatment naïve (%)	84 (71.2%)	59 (71.1%)	0.987
Off-treatment follow-up period (y.)	2.36 ± 1.14	2.04 ± 1.13	0.054

ALT, alanine aminotransferase; HBsAg, hepatitis B surface antigen; HBV, hepatitis B virus Continuous variables are expressed as the mean ± standard deviation or median (range).

^*^One hundred and three HBeAg-positive and sixty HBeAg-negative CHB patients had a baseline qHBsAg level.

**Table 2:** Univariate and multivariate analysis of baseline factors associated with 48 weeks of off-treatment HBe seroconversion and combined response among HBeAg-positive chronic hepatitis B.

HBeAg-positive CHB patients (N = 118)								
	HBe seroconversion*				Combined response*			
	Univariate		Multivariate		Univariate		Multivariate	
	OR (95% CI)	*p*	OR (95% CI)	p	OR (95% CI)	p	OR (95% CI)	p
Age (year)	0.96 (0.92 ~ 1.01)	0.158		NA	0.98 (0.94 ~ 1.04)	0.536		NA
Sex (male) (n/N=84/118)	2.18 (0.85 ~ 5.61)	0.105		NA	0.7 (0.25 ~ 1.92)	0.492		NA
Treatment naïve (%) (n/N = 84/118)	0.71 (0.29 ~ 1.72)	0.444		NA	0.96 (0.36 ~ 2.56)	0.931		NA
Treatment duration (weeks)	0.96 (0. 91 ~ 1.01)	0.136		NA	0.93 (0.85 ~ 1.02)	0.115		NA
ALT ≥200 U/L (n/N = 48/118)	2.53 (1.08 ~ 5.9)	0.032	2.46 (1.04 ~ 5.86)	0.042	3.37 (1.31 ~ 8.72)	0.012	2.44 (0.95 ~ 6.25)	0.063
HBsAg <250 IU/mL (n/N = 33/103)	2.42 (0.98 ~ 5.97)	0.054		NA	2.288 (1 ~ 5.23)	0.05	2.67 (1.01 ~ 6.49)	0.03
HBsAg <1,250 IU/mL (n/N = 45/103)	1.42 (0.55 ~ 3.65)	0.471		NA	4.57 (0.56 ~ 37.55)	0.157		NA
HBsAg <25,000 IU/mL (n/N = 93/103)	10.45 (1.34 ~ 81.6)	0.025	7.93 (0.99 ~ 63.68)	0.051	6.41 (0.82 ~ 50.44)	0.078		NA
HBV DNA <2.5 × 10^7^ (IU/mL) (n/N = 59/118)	1.3 (0.6 ~ 2.84)	0.51		NA	2.66 (1.04 ~ 6.78)	0.041	2.87 (1.27 ~ 6.5)	0.011

OR, Odds ratio; CI, confidence interval; NA, not adopted; NS, not significant; ALT, alanine aminotransferase; HBsAg, hepatitis B surface antigen; HBV, hepatitis B virus.

^*^Among 118 HBeAg-positive CHB patients, HBeAg seroconversion rate and the combined response rate were 31.4% (37/118) and 21.2% (25/118) at 48 weeks after EOT.

**Table 3:** Two models for predicting sustained off-treatment combined response at 48 weeks after EOT among HBeAg-positive chronic hepatitis B patients.

HBeAg-positive CHB patients	
Factors	points
Model A	
HBsAg <250 IU/mL	1
HBV DNA <2.5 × 10^7^ IU/mL	1
Model B	
HBsAg <250 IU/mL	2
HBV DNA <2.5 × 10^7^ IU/mL	2
ALT ≥200 U/L	1

HBsAg, hepatitis B surface antigen; HBV, hepatitis B virus; ALT, alanine aminotransferase.

**Table 4:** Univariate and multivariate analysis of baseline factors associated with sustained off-treatment virological response among HBeAg-negative chronic hepatitis B patients*.

HBeAg-negative CHB patients (N = 83)				
	Univariate		Multivariate	
	OR (95% CI)	*p*	OR (95% CI)	*p*
Age (year)	0.98 (0.94 ~ 1.04)	0.53		NA
Sex (male) (n/N = 71/83)	0.72 (0.14 ~ 3.7)	0.698		NA
Treatment naive (%) (n/N = 59/83)	0.21 (0.06 ~ 0.69)	0.011	0.36 (0.04 ~ 2.99)	0.343
Treatment duration (weeks)	0.65 (0.34 ~ 1.24)	0.192		NA
ALT ≥200 U/L (n/N = 23/83)	1.2 (0.42 ~ 3.46)	0.732		NA
HBsAg <250 IU/mL (n/N = 10/60)	1.06 (0.18 ~ 6.29)	0.948		NA
HBsAg <1,250 IU/mL (n/N = 22/60)	6.13 (1.09 ~ 34.35)	0.039	10.39 (5.96 ~ 55.04)	0.006
HBV DNA <2.5 × 10^7^ (IU/mL) (n/N = 72/83)	4.4 (0.53 ~ 36.51)	0.17		NA

OR, Odds ratio; CI, confidence interval; NA, not adopted; ALT, alanine aminotransferase; HBsAg, hepatitis B surface antigen; HBV, hepatitis B virus.

^*^Among 83 HBeAg-negative CHB patients, sustained off-treatment virological response rate was 27.7% (23/83) at 48 weeks after EOT.
