# Supplementary material for: Predictors of response to pegylated interferon in chronic hepatitis B: a real-world hospital-based analysis
Source: Sci Rep. 2016 Jul 12;6:29605. doi: 10.1038/srep29605 (PMC4941731; doi:10.1038/srep29605)
Supplement: Supplementary Information [file srep29605-s1.pdf]

Supplementary materials:

Predictors of response to pegylated interferon in chronic hepatitis B: a real-world hospital-based analysis

Yin-Chen Wang[1], Sien-Sing Yang[2*], Chien-Wei Su[1], Yuan-Jen Wang[3], Kuei-Chuan Lee[1], Teh-Ia Huo[1,4], Han-Chieh Lin[1], Yi-Hsiang Huang[1,5*]

[1]Division of Gastroenterology and Hepatology, Department of Medicine, Taipei Veterans General Hospital, Taipei, Taiwan; [2] Liver Center, Cathay General Hospital Medical Center, Taipei, Taiwan; [3] Health Care Center, Taipei Veterans General Hospital, Taipei, Taiwan; [4]Institute of Pharmacology, National Yang-Ming University School of Medicine, Taipei, Taiwan; [5]Institute of Clinical Medicine, National Yang-Ming University, Taipei, Taiwan

Supplementary Table S1. Univariate and multivariate analysis of factors associated with 48 weeks of off-treatment combined response among HBeAg-positive chronic hepatitis B patients with available on-treatment HBsAg data

HBeAg-positive CHB*		
	Univariate<br>OR (95% CI)	p
**Baseline data**		
Age (year)	1.01 (0.91~1.12)	0.85
Sex (male)	0.58 (0.05~7.43)	0.678
Treatment naive (%)	1.22 (0.1~15.23)	0.876
Treatment duration (weeks)	0.99 (0.89~1.1)	0.813
Genotype (B vs. non-B)	2.1 (0.~2.15 x10^8)	0.999
ALT ≥ 200 U/L	2.77 (0.23~33.88)	0.425
HBsAg < 250 IU/mL	3.07 (0~2.69x10^8)	0.999
HBV DNA < 2.5x10^7 IU/mL	6.25 (0~4.85x10^8)	0.998
**On treatment**		
HBsAg decline ≥ 10%	1.1 (0.09~13.55)	0.941
HBsAg decline ≥ 1 log†	-	-

OR, odds ratio; CI, confidence interval; ALT, alanine aminotransferase; HBsAg, hepatitis B surface antigen; HBV, hepatitis B virus

* 34 HBeAg-positive CHB patients had available on-treatment HBsAg levels

† Only three cases achieved the on-treatment HBsAg decline ≥1 log; logistic regression models were failed to analyze due to small case numbers.

Supplementary Table S2. Univariate and multivariate analysis of factors associated with 48 weeks of off-treatment response among HBeAg-negative chronic hepatitis B patients with available on-treatment HBsAg data

HBeAg-negative CHB*				
	Univariate<br>OR (95% CI)	p	Multivariate<br>OR (95% CI)	p
Baseline data				
Age (year)	0.97 (0.90~1.05)	0.515		NA
Sex (male)	0.86 (0.21~3.49)	0.84		NA
Treatment naive (%)	0.13 (0.02~1.02)	0.052	0.37 (0.02~6.89)	0.507
Treatment duration (weeks)	0.68 (0.36~1.27)	0.224		NA
Genotype (B vs. non-B)	1.8 (0.3~10.9)	0.522		NA
ALT ≥ 200 U/L	2 (0.4~9.91)	0.396		NA
HBsAg < 250 IU/mL	1.06 (0.18~6.29)	0.948		NA
HBsAg < 1,250 IU/mL	5.1 (0.86~30.27)	0.073	2.22 (0.22~22.14)	0.496
HBV DNA < $2.5x10^7$ IU/mL	2.15 (0.23~20.23)	0.502		NA
On treatment				
HBsAg decline ≥ 10%	8.75 (0.94~81.26)	0.056		NA
HBsAg decline ≥ 1 log	37.5 (4.36~322.9)	0.001	25.83 (2.77~241.08)	0.004

OR, Odds ratio; CI, confidence interval; NA, not adopted; ALT, alanine aminotransferase; HBsAg, hepatitis B surface antigen; HBV, hepatitis B virus

* 35 HBeAg-negative CHB patients had available on-treatment HBsAg levels

Supplementary Table S3. Univariate and multivariate analysis of the factors associated with relapse among HBeAg-negative chronic hepatitis B patients with virological response at EOT

	Univariate HR (95% CI)	p	Multivariate HR (95% CI)	p
**Baseline data**				
Age (year)	1.03 (0.98~1.09)	0.303		NA
Sex (male)	1.45 (0.27~7.69)	0.663		NA
Treatment naive	4.35 (1.45~14.29)	0.009	6.25 (0.73~52.63)	0.125
Not using IFN	5.88 (1.01~33.33)	0.049		NA
Not using NAs	2.63 (0.76~9.09)	0.126		NA
Treatment duration (weeks)	1.05 (0.6~1.85)	0.858		NA
ALT ≥ 200 U/L	0.87 (0.29~2.55)	0.792		NA
HBV DNA (log IU/mL)	1.05 (0.74~1.48)	0.791		NA
HBV DNA ≥ $2.5x10^7$ (IU/mL)	3.57 (0.42~34.48)	0.246		NA
HBsAg ≥ 1,250 IU/mL	6.67 (1.16~33.33)	0.034	7.69 (1.37~50.55)	0.02
**On-treatment**				
HBsAg decline < 10% from baseline*	5.59 (0.56~46.95)	0.145		NA
HBsAg decline < 1 log from baseline*	35.71 (3.57~253.16)	0.002		NA
**End of treatment**				
ALT ≤ 40 U/L	1.56 (0.56~4.39)	0.395		NA
HBV DNA (log IU/mL)	1.31 (0.83~2.07)	0.242		NA
HBV DNA detectable (≥ 12 IU/mL)	3.57 (1.04~12.5)	0.043	19.6 (1.33~103.1)	0.031
HBsAg ≥ 200 IU/mL†	6.25 (1.06~35.71)	0.042		NA
HBsAg ≥ 100 IU/mL†	4.35 (0.88~21.28)	0.071		NA

HR, hazard ratio; CI, confidence interval; NA, not adopted; ALT, alanine aminotransferase; HBV, hepatitis B virus; HBsAg, hepatitis B surface antigen; * Total 35 HBeAg-negative CHB patients had available on-treatment HBsAg levels

† Total 28 HBeAg-negative CHB patients had a qHBsAg level at EOT

Supplementary Table S4. Scores for individual factors associated with relapse in HBeAg-negative chronic hepatitis B patients achieving virological response at EOT

HBeAg-negative CHB patients	
**Model A**	
factors	points
Baseline HBsAg ≥ 1,250 IU/mL	1
HBV DNA detectable (≥ 12 IU/mL) at EOT	1
**Model B**	
factors	points
Baseline HBsAg ≥ 1,250 IU/mL	1
On-treatment HBsAg decline <1 log from baseline	1
HBV DNA detectable (≥ 12 IU/mL) at EOT	1
HBsAg ≥ 200 IU/mL at EOT	1

HBsAg, hepatitis B surface antigen; HBV, hepatitis B virus; EOT, end-of-treatment

Supplementary Figure S1. Two models for predicting relapse rates at 48 weeks after EOT among responders at EOT in HBeAg-negative chronic hepatitis B patients*

The relapse rates among HBeAg-negative chronic hepatitis B patients according to models: (A) model A, (B) model B

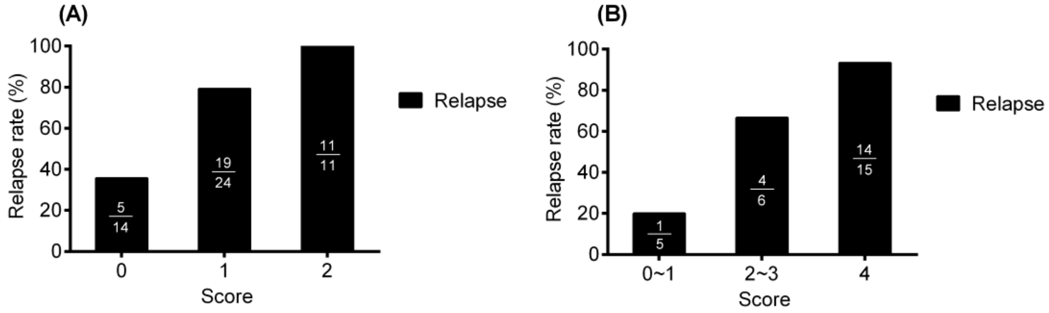

* Forty nine HBeAg-negative CHB patients who had baseline qHBsAg level and available HBV viral load at end-of-treatment. Among these patients, 26 patients had on-treatment qHBsAg level.
